# Dealing with cultural diversity during the process of communication and decision-making in the ICU: a literature review

**DOI:** 10.1186/cc13214

**Published:** 2014-03-17

**Authors:** RV Van Keer, RD Deschepper, LH Huyghens, WD Distelmans, JB Bilsen

**Affiliations:** 1 Vrije Universiteit Brussel, Brussels, Belgium

## Introduction

Studies have shown that communication in the ICU is related to the cultural background of all involved actors. As a consequence, hospitals in multiethnic areas in western countries are currently forced to rethink their models of communication as they are increasingly transformed into spaces of cultural encounter. During this process one needs to be aware of the cultural differences in attitudes and experiences towards communication in critical medical situations. However, the growing academic interest in intercultural relations in the critical care has not yet led to the construction of cumulative knowledge. The aim of this study is to review the experiences of the involved actors, namely the care providers, the patients and their family members, with cultural diversity during the process of communication and decision-making in the ICU.

## Methods

A literature review was conducted. PubMed was searched. The following combinations of Mesh terms were used: [(decisionmaking) OR (communication)] AND (intensive care) OR (intensive care units)] AND [(cultural diversity) OR (multiculturalism) OR (migrants) OR (culture) OR (ethnicity)]. A total of 111 studies were retrieved. We excluded nonempirical studies, studies not written in English and articles published before 2003. After screening the titles and abstracts 12 articles were selected. The full text of these articles was analyzed and synthesized with a thematic-synthesis approach. During the process we performed additional searches based on the bibliographies of all the relevant articles.

## Results

Culturally specific expectations about communication and decision-making, different views on gender roles, organization of the care and expression and resolving emotional and social crises may cause extra stress. The communication becomes more complex when the language of the care providers differs from the language of the patient and family. Intercultural communication can be improved by specific actions such as ethnic matching, using an interpreter and the formation of cultural-competent healthcare professionals.

## Conclusion

There is a lack of knowledge regarding the specific experiences of patients, families and care providers with communication and especially decision-making in multicultural ICUs in Western Europe.

